# The effect of Baduanjin exercise for physical and psychological wellbeing of college students: study protocol for a randomized controlled trial

**DOI:** 10.1186/1745-6215-14-422

**Published:** 2013-12-05

**Authors:** Guohua Zheng, Moyi Li, Xiulu Lan, Xinghui Yan, Qiu Lin, Lidian Chen, Jing Tao, Xin Zheng, Junzhe Li, Bai Chen, Qianying Fang

**Affiliations:** 1 Academy of Integrative Medicine, Fujian University of Traditional Chinese Medicine, Fuzhou 350122, China; 2 Rehabilitation Medicine College, Fujian University of Traditional Chinese Medicine, Fuzhou 350122, China; 3 Department of Physical Education, Fujian University of Traditional Chinese Medicine, Fuzhou 350122, China; 4 Fujian University of Traditional Chinese Medicine, Fuzhou 350122, China

**Keywords:** Baduanjin exercise, College students, Physical health, Mental health, Randomized controlled trial

## Abstract

**Background:**

The physical and mental health of college students tends to continuously decline around the world. Since they are in a significant transition period which presents opportunities and challenges in health promotion, it is important to improve their health in this period. As a traditional Chinese exercise form which combines movements with breath and mind, Baduanjin may be one of the selectable effective exercises. However, evidence of Baduanjin exercise for college students has not been completely established. The primary aim of this trial is to evaluate the effectiveness and safety of Baduanjin exercise for physical and mental health of college students through a rigorous randomization, parallel-controlled design.

**Method/design:**

We will conduct a randomized, single-blind, parallel-controlled trial. A total of 222 college students from Fujian University of Traditional Chinese Medicine who meet the eligibility criteria will be recruited and randomly allocated into Baduanjin training or usual exercise control group. Baduanjin training will last 12 weeks (1 h per day, 5 days per week). The physical and psychological outcomes, including lumbar muscle strength, lumbar proprioception function, physical fitness, as well as self-reported symptom intensity, stress, self-esteem, mood, quality of life, quality of sleep, and adverse events, will be evaluated by blinded outcome assessors at baseline, 13 weeks (at the end of intervention), and 25 weeks (after the 12-week follow-up period).

**Discussion:**

This protocol presents an objective design of a randomized, single-blind trial that aims to evaluate the effectiveness and safety of Baduanjin exercise for physical and mental health of college students. If the outcome is positive, the results will provide higher-quality evidence to better inform the college students regarding their selection about whether to receive such exercise.

**Trial registration:**

Chinese Clinical Trial Registry: ChiCTR-TRC-13003329

Registration date: 18 July, 2013.

## Background

College or university students are in a key transition period of growth and development that connects high school with adulthood [1]. In 2012, the general tertiary education enrollment in China had reached 23.913 million students with more than 6.88 million new students, most of whom were young adults aged 18 to 25 years [2]. Young adults in this period will experience many rapid changes in the body, mind, and social relationships [3]. They will complete the transition to autonomy in making decisions and developing behavior patterns independently, in particular health behaviors that will often continue throughout their lives [4]. Young adults are generally thought to be in good health; their health problems, therefore, do not need more attention [5]. Although risk factors and lifestyles that young adults adopt might not affect their health during this period, they can have a substantial effect in later life and future generations [6]. Studies indicated that the levels of physical inactivity among college students were increasing gradually around the world due to lifestyle changes and behavior habits, lower study pressure, and pervasive presence of the Internet [7-10]. It is estimated that 40% to 50% of U.S. college students are physically inactive [11,12]. Data from the Behavioral Risk Factor Surveillance System telephone survey shows 43% of respondents aged 18 to 24 years reported insufficient amounts or no physical activity [13]. Similarly, the percentage of physically inactive students was 13.5% for Taiwan, 16.8% for Hong Kong, and 28.5% for Korea [14]. Furthermore, physical inactivity has been demonstrated to have independent association with the increased risk of weight gain, metabolic syndrome, diabetes, and heart disease [15]. College students may therefore be a population at risk and susceptible to chronic diseases [16-18].

On the other hand, as a result of risky behaviors and multiple stressors, such as academic challenge, achievements, and competition with peers [19], various forms of psychological problems are more frequently present among college students than non-student populations of the same age [20]. These disorders appear to be increasing in number and severity [21,22]. Mental disorders were reported accounting for nearly half of the disease burden for young adults in the U.S. [23]. One survey among 1,027 U.S. medical students showed 47% of students reported at least one mental health concern, 26% of the total reported stress, 19% reported anxiety, and 18% reported depression [24]. The longitudinal studies of psychological distress also showed that although distress levels peaked during the first year and then declined for most students, some of them manifested severe psychological distress and did not decrease over time [25,26]. If left ignored and untreated, these mental health problems may lead to students dropping out or failing out of college, attempting or committing suicide, or engaging in other risky, dangerous behavior [27]. However, it is estimated that only a minority of college students with mental health problems seek and receive adequate help [28].

As college students are in such a significant transition period in their lives which presents opportunities and challenges in improving health [29], it is urgent to promote their physical and mental health.

The growing evidence continues to support the idea that regular exercise or physical activity are positively associated with physical and psychological health outcomes [30]. Recent studies showed that the behavioral exercise programs for 8 to 12 weeks were effective for the promotion of the body composition, physical fitness, and the mental health condition in college students [31-33]. As a traditional Chinese mind-body aerobic exercise, Qigong is based on Taoist philosophy and traditional Chinese medicine theories [34]. Qigong is a combination of postures, meditation, and movements designed to improve holistic health and to facilitate mind-body integration [34,35]. It has a history of several thousand years and is a highly popular practice, particularly in China, for health maintenance, healing, and increasing vitality [36,37].

Baduanjin exercise, translated as the ‘eight section of brocades’, is one of the most common forms of Qigong. Baduanjin exercise consists of eight separate, delicate, and smooth exercise movements, in which each section brings certain function-enhancing benefits to different physical parts of body or particular organs [38]. For example, the first section helps regulate all internal organs through moving hands up and down over the head [39]. Baduanjin exercise can enhance Qi function through the whole exercise of body posture, movement, breathing, and meditation - that is, to draw upon natural forces to optimize and balance energy within, through the purposeful coordination of body, breath, and mind [38]. With the combination of self-awareness with self-correction of the posture and movement of the body, the flow of breath, and stilling of the mind, Baduanjin exercise is thought to comprise a state which activates the natural self-regulation capacity, stimulates the balanced release of endogenous neurohormones, and a wide array of natural health recovery mechanisms [40]. Current studies have suggested that Baduanjin training appears to have substantive benefits for older adults with some physical and mental disorders such as anxiety, obsessive-compulsive symptoms, depression, hyperlipidemia, spinal problems, osteoarthrosis, and type 2 diabetes [41-43]. For young adults, particularly the college student population, the results from few studies also indicated that Baduanjin exercise has a potential benefit on reducing depression, stress and anxiety, building self-control and a healthy mind, and improving physical function [44-46]. However, the evidence is unclear whether Baduanjin exercise can be recommended as an effective exercise to improve psychological wellbeing, and physical fitness due to the limited methodological quality in their studies. The purpose of this trial is to systematically evaluate the effects of Baduanjin exercise on physical and psychological outcomes of the college students including lumbar muscle strength, lumbar proprioception function, physical fitness, self-reported symptom intensity, stress, self-efficacy, attention, mood and mindfulness, self-esteem, quality of life, and quality of sleep.

## Method/design

### Study objective

To assess the effectiveness and safety of Baduanjin exercise on physical and psychological health of college students.

### Study design

**Figure 1:**
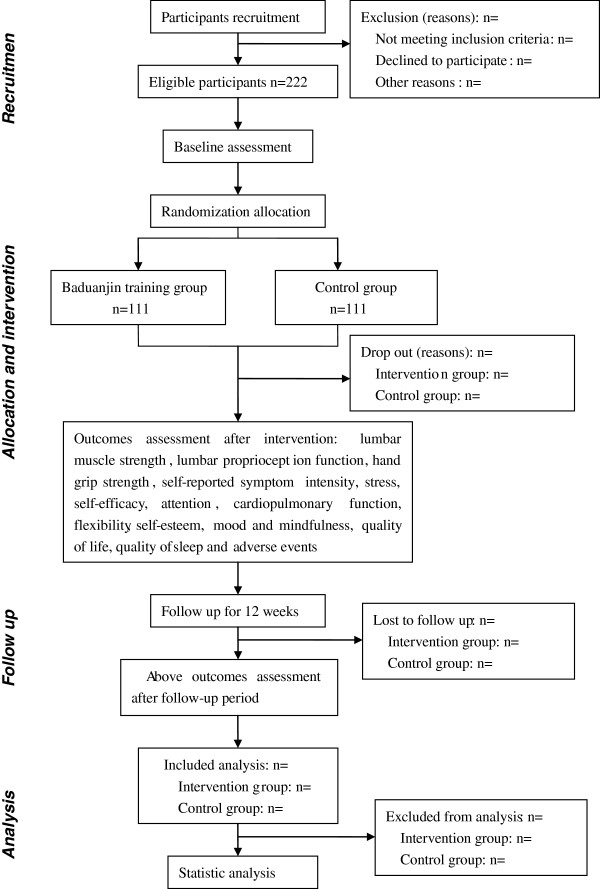
Flow diagram of participants.

A randomized, single-blind, parallel-controlled trial will be conducted to evaluate the effectiveness and safety of Baduanjin exercise for the physical and mental health of college students. A total of 222 eligible college students from the Fujian University of Traditional Chinese Medicine (FJTCM) will be recruited and randomly allocated to either the Baduanjin exercise or usual exercise control group. The relative physical and psychological outcomes will be measured at baseline, 13 weeks (at the end of intervention), and 25 weeks (after the 12-week follow-up period). The flow diagram for this trial is presented in Figure 1.

### Sample size

We use the improvement of lumbar flexion muscle strength as the main effect indicators to estimate the sample size. As no previous study is available, our preliminary test data from 20 college students with usual exercise indicated the means with standard deviation of the lumbar flexion muscle strength was 258.25 newton (N) and 114.19 N, respectively, to detect a 20% mean difference on the Baduanjin exercise at end of intervention. One hundred and one individuals per group are calculated according to the formula $n_{1}=n_{2}=2{\left(\frac{Z_{\alpha /2}+Z_{\beta }}{M_{e}-M_{c}}\right)}^{2}\delta^{2}$ with a type I error of 5% (α = 0.05) and 90% power (β = 0.10). Assuming a dropout rate of 10% for follow-up [47,48], the sample size is 111 for each group and 222 in the two groups.

### Participant and recruitment

We will recruit 222 college students from FJTCM who are in the first or second grade aged 18 to 25 years. The eligible participants should fulfil the following inclusion criteria and not comply with the exclusion criteria. Recruitment of participants will be performed at the campus of FJTCM by schoolyard advertisement and school radio. Those who are interested in taking part will contact the screeners who will determine eligibility at the recruitment office. If an applicant meets the study criteria, he or she will be invited to participate in the research.

### Inclusion criteria

For inclusion, participants should: be aged 18 to 25 years; be able to give the informed consent form; and be a full-time student at first or second grade.

### Exclusion criteria

Criteria for exclusion from the study are as follows: being or having been engaged in a long-term regular practice of Baduanjin; being a member of the Martial Arts Association, Dance Association, Aerobics Association, Sanda Association, or Taekwondo Association; those who have suffered from severe cardiovascular diseases, musculoskeletal system diseases, or other sports contraindications.

### Randomization and allocation concealment

The random allocation sequence will be produced by an independent statistician via the PLAN sentences of the statistical software SAS9.1, who works in the Evidence-Based Center of FJTCM. The eligible participants will be allocated to either Baduanjin exercise or usual exercise control group according to 1:1 equal proportion rule. The random allocation sequence will be managed by a specified project manager who is not involved in the recruitment program of this trail, and be concealed to the screeners and outcome assessors. The eligible participants will be informed their allocation result by the project manager via telephone.

### Blinding

In this trial, it is impossible to blind the participants and exercise coaches. We will assign a specified project manager to be in charge of the management of the random allocation sequence and blind code of allocation in which the intervention group (Baduanjin exercise or usual exercise control group) will be replaced by the alphabet A or B. Furthermore, we will rule that each investigator has a well-defined obligation: the project manager and exercise coaches will be not involved in the assessment of outcome; and the outcome assessors and the statistic analyzer will be not involved in the participants’ screening and allocating. The blind code will be disclosed when the statistic analysis is completed.

### Intervention

#### Baduanjin exercise group

**Figure 2:**
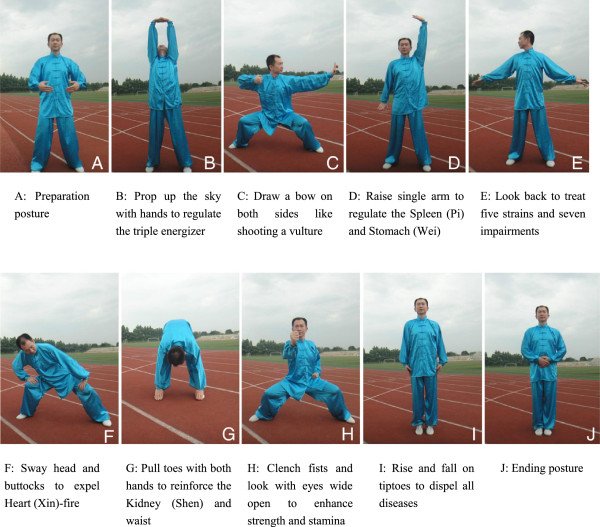
**Ten postures of Baduanjin. (A)** Preparation posture. **(B)** Prop up the sky with hands to regulate the triple energizer. **(C)** Draw a bow on both sides like shooting a vulture. **(D)** Raise single arm to regulate the spleen (Pi) and stomach (Wei). **(E)** Look back to treat five strains and seven impairments. **(F)** Sway head and buttocks to expel heart (Xin)-fire. **(G)** Pull toes with both hands to reinforce the kidney (Shen) and waist. **(H)** Clench fists and look with eyes wide open to enhance strength and stamina. **(I)** Rise and fall on tiptoes to dispel all diseases. **(J)** Ending posture.

The Baduanjin exercise training will be applied to the participants in the Baduanjin exercise group, in which the Baduanjin exercise training will be performed at the gymnasiums of the university. The training scheme originated from the *Health Qigong - Baduanjin*, published by the General Administration of Sport of China which is the most popular style in the Chinese population [49]. The whole set of Baduanjin exercise consists of 10 postures (including the beginning and ending posture) (Figure 2). Two qualified coaches who have engaged in the physical education over 5 years will teach the participants the correct Baduanjin postures during the whole intervention period.

#### Control group

The participants in the control group will not receive any specific training from the study scheme. They will be informed keeping their original exercises.

### Intervention regimen

The intervention period in this trial will last 12 weeks. Participants in the Baduanjin exercise group will undergo the regular Baduanjin exercise training at a frequency of 5 days a week with 1 h per day. All participants will be required to record their daily activity or sport information during the intervention period.

### Follow-up period

During the 12-week unsupervised follow-up period, no participants will undergo special exercise intervention with the exception of their usual exercises. However, all participants will be required to record their daily activity or exercise information. The primary and second outcomes will be re-measured after the 12-week follow-up period.

### Outcome assessment

Outcome measurements consist of lumbar muscle strength, lumbar proprioception function, physical fitness (including hand grip strength, cardiopulmonary function, and flexibility), as well as self-reported symptom intensity, mood, self-efficacy, attention, quality of life, and quality of sleep, and so on. All primary and secondary outcomes will be assessed at baseline, 13 weeks (end of intervention), and 25 weeks (after the 12-week follow-up period). Lumbar muscle strength and lumbar proprioception function will be assessed by experienced operators at the Evaluation Department of Rehabilitation Hospital Affiliated to FJTCM. Physical fitness tests will be performed by physical education teachers at gymnasium according to the *Chinese University Students’ Physical Health Standards*. Several outcome assessors who are in charge with the psychological measurement will investigate the psychological status of the participants (such as General Self-efficacy Scale, Symptom Checklist-90, and Chinese Perceived Stress Scale) at College Student Activity Center. A summary of all measures in the trial is shown in Table 1.

**Table 1:** Trial processes chart

**Items**		**Before enrollment (week −2-(−1)**	**Intervention period (weeks 1–12)**	**End of intervention (week 13)**	**Follow-up period (weeks 13–24)**	**End of follow-up (week 25)**
Inclusion criteria		×				
Exclusion criteria		×				
Informed consent		×				
Baseline measurement		×				
Randomization and allocation		×				
Lumbar muscle strength		×		×		×
Lumbar proprioception function		×		×		×
Self-reported symptom intensity		×		×		×
Stress		×		×		×
Self-efficiency		×		×		×
Attention		×		×		×
Physical fitness	Cardiopulmonary function	×		×		×
	Flexibility	×		×		×
	Hand grip strength	×		×		×
Self-esteem		×		×		×
Mood and mindfulness		×		×		×
Quality of life		×		×		×
Quality of sleep		×		×		×
Adverse events			×		×	
Self-report diaries			×		×	

### Primary outcomes

• Lumbar muscle strength consists of flexion, extension, lateroflexion, and rotation myodynamia. It will be assessed with the *Tergumed Work Station* produced by Proxomed GmbH, Germany (product type: Flexion Work Station:Tergumed Flexion; Lateral Flexion Work Station: Tergumed lateral Flexion; Extension Work Station: Tergumed Extension; Rotation Work Station: Tergumed Rotation).

• Lumbar proprioception function will be measured by the *Prokin* proprioception evaluation and training system (product type: PK254P) produced by Tecnobody .S.r.l, Italy.

• The Symptom Checklist-90 (SCL-90) will be used to measure symptom intensity [50], which consists of the following 10 symptom factors: somatization, obsessive-compulsive disorder, interpersonal sensitivity, depression, anxiety, hostility, phobic anxiety, paranoid ideation, psychoticism, and other.

• Stress will be measured with the Chinese version of the Perceived Stress Scale revised by Yang Tingzhong and Huang Hanteng [51] - Chinese Perceived Stress Scale (CPSS). The scale consists of 14 questions that reflect uncontrollability and tension of stress. Participants are asked to respond to each question on a 5-point Likert scale ranging from 0 (never) to 4 (very often). Higher composite scores indicate greater perceived stress.

• Self-efficacy will be assessed with General Self-efficacy Scale (GSES). We will use the Chinese version translated by Zhang Jianxin and Schwarzer [52], which includes 10 questions, and responses are on a 4-point Likert scale ranging from 1 (completely incorrect) to 4 (completely correct), with higher composite scores indicative of greater self-efficacy.

• Attention will be assessed by Schulte Grid (8*8) test. Schulte Grid (8*8) is a square that consists of 64 squares of the same size (1 × 1 cm), with one of 64 random numbers from 1 to 64. When tested, individuals are required to figure out the numbers in the order from 1 to 64, and read out the numbers loud at the same time. Timing starts with 1 and ends with 64. Less time represents higher level of attention.

### Secondary outcomes

• Physical fitness includes cardiopulmonary function, flexibility, and hand grip strength.

1. Cardiopulmonary function will be assessed by the step test, vital capacity, blood pressure, and heart rate. The step test will be measured by step testers produced by Zhongtitongfang Co., Ltd., Beijing (product type: CSTF-TZ-5000). The vital capacity will be measured by vital capacity testers produced by Zhongtitongfang Co., Ltd., Beijing (product type: CSTF-FH-5000). While blood pressure and heart rate will be tested by electric sphygmomanometers produced by the Omron Corp., China (product type: HEM-746C).

2. Flexibility will be measured by the ‘sit and reach’ test with a ‘sit and reach’ tester produced by Zhongtitongfang Co., Ltd., Beijing (product type: CSTF-TQ-5000).

3. Hand grip strength will be measured by a hand grip strength tester produced by Zhongtitongfang Co., Ltd., Beijing (product type: CSTF-WL-5000).

• The Self-Esteem Scale (SES) will be administered to measure self-esteem. We will use the Chinese version of SES translated by Ji Yifu and Yu Xin [53]. It consists of 10 items, and the total score ranges from 10 to 40. Higher scores indicate higher self-esteem.

• Mood and mindfulness will be measured by Profile of Mood States (POMS). The version we will use is translated and revised by Zhu Beili in 1995 [54]. It contains seven subscales (tension, anger, fatigue, depression, vigor, confusion, and mood related to self-esteem) with 40 adjectives that describe mood. Higher POMS Total Mood Disturbance (TMD) scores stand for more negative current mood states.

• Quality of life will be measured by the Chinese version of the World Health Organization Quality of Life - BREF (WHOQOL-BREF) translated by Fang Jiqian et al. [55]. It consists of 26 items, and 24 of them are categorized into four domains: physical capacity (7 items), psychological wellbeing (6 items), social relationships (3 items), and environment (8 items). The other two items measure overall QOL and general health [56].

• Quality of sleep will be measured by the Pittsburgh Sleep Quality Index (PSQI) [57]. The Chinese version of PSQI has been reported acceptable internal consistency, test-retest reliability, construct validity, and criterion-related validity by Liu Xianchen and his colleagues [58]. Nineteen individual items generate seven component scores: subjective sleep quality, sleep latency, sleep duration, habitual sleep efficiency, sleep disturbances, use of sleeping medication, and daytime dysfunction. The sum of the seven component scores ranges from 0 to 21; higher scores represent poorer subjective sleep quality [59].

### Safety measurements

As far as we know, there are no adverse events (AEs) reported about Baduanjin exercise. However, all unexpected AEs related to Baduanjin will be reported to the researchers or the project manager if they happen. The AEs will be analyzed regardless of the investigators’ assessments of causality. If serious AEs happen, the researchers will report to the primary investigator and ethics committee immediately, who will make a decision on whether the participant needs to withdraw from the study.

### Data collection and management

The demographic and baseline characteristic data will be collected by screeners when the participants are recruited. Primary and secondary outcome will be measured by the outcome assessors at baseline, 13 weeks (end of intervention), and 25 weeks (after the 12-week follow-up period). Research assistants will conduct quality control of data collection and be responsible for data entry. The project manager will be responsible for initial data cleaning, identifying, coding, and converting into the proper format for data analysis.

### Statistical analysis

Analysis of all data in this trial will be performed by a statistician who is not involved in this trial at the Center of Evidence-based Medicine, Fujian University of Traditional Chinese Medicine.

In descriptive analysis of the sample, continuous variables will be expressed by using mean and standard deviation for normal distribution, and median and interquartile range for non-normal distribution. Normality will be tested using Kolmogorov-Smirnov test. Appropriate transformations will be applied in cases of non-normal distribution. Categorical variables will be expressed as proportions with their standard error.

Baseline characteristics between groups will compare using the *t*-test or Mann–Whitney test for continuous variables and Pearson chi-squared or Fisher’s exact test for categorical variables. If incomparability appears, the inequality factors will be treated as confounding variables in the final efficacy analysis.

For comparison of the primary or secondary outcomes between groups, a *t*-test or non-parametric tests will be used for continuous data, and Pearson chi-squared or Fisher exact test for categorical data. To control for possible confounding variables, linear models or linear regression will be applied for dependent continuous variables and logistic regression models for dependent categorical variables. Subgroup analysis stratified by participants’ sex will be used for the primary outcomes. Analysis of variance (ANOVA) will be used for the repeated measurement data, and the *post-hoc* comparison will be applied if the difference is found significant. Analysis of the primary and secondary outcomes will be on the basis of the intention-to-treat (ITT) population and per-protocol (PP) population. The result of the ITT analysis will be compared with that of the PP analysis to determine whether the results are consistent. Missing data will be completed by Last Observation Carry Forward rules.

AEs will be listed and analyzed using a Chi-square test or Fisher’s exact test. Severe AEs will be listed in detail. All data will be analyzed with SPSS 21.0 (IBM, Chicago, IL, USA) software packages. The statistical significance is defined as two-sided *P* value of <0.05.

### Ethics issue

This protocol is conducted in accordance with the Declaration of Helsinki. The study protocol and consent forms were approved by the Ethics Board of FJTCM (approval number: 2013, Reviewed-No. 043). All participants will be fully informed about the trial, and will sign the informed consent form prior to participation.

## Discussion

Baduanjin exercise is one of the most common forms of Chinese traditional exercise. It is broken down into eight separate sections, and each one focuses on a different physical area and Qi meridian [38]. As an aerobic exercise, Baduanjin is not only easy to learn, but it also has a less cognitive demanding [60]. Therefore, it has been taken as a popular community exercise to promote health in China [61]. Previous studies have indicated that a Baduanjin exercise program can improve blood lipid metabolism, insulin sensitivity, and sleep quality for community older adults [39,41-43], but there is currently a lack of evidence regarding the associations between Baduanjin exercise and physical fitness, as well as self-reported symptom intensity, mood, self-esteem, and self-efficacy. In addition, from a sports medicine point of view, Baduanjin exercise focuses on lumbar function, and stresses ‘take the waist as the axis’ when practicing [62]. It is therefore expected that Baduanjin exercise enhances lumbar muscle strength and lumbar proprioception function. However, related research is not found. In this trial, we will apply modern devices including the *Tergumed Work Station* and the *Prokin* proprioception evaluation system to measure the change of lumbar muscle strength and lumbar proprioception function. This trial focuses on a college student population. Through a 12-week intervention with Baduanjin exercise, results from a range of primary and secondary outcome measures will provide the clear information about difference in physical and psychological outcomes between Banduanjin exercise and usual exercise control groups. In this trial, we performed the rigorous randomized, parallel-controlled design with a large sample (*n* = 222) to evaluate the effectiveness and safety of Baduanjin exercise. We will arrange two qualified physical teachers to serve as the Baduanjin exercise coaches in order to ensure the standardized exercise training for participants. Participants in Baduanjin exercise group will be gathered to do the exercise at a fixed setting and time. To control the trial bias, all participants will be required to record their physical activity and sport diary. Furthermore, the result evaluators and statistical analysts will be blinded to ensure the authenticity and objectivity of the trial results.

Several potential limitations may occur in this trial. Ideally, everyone involved in an RCT should be blinded, but this is not always feasible in the non-pharmacological trials [63]. Although the participants and exercise coaches are not blinded and the psychological outcome measures are participants’ self-reported, the assessment of the related physical fitness outcomes, lumbar muscle strength, lumbar proprioception function, and the statistical analyses will be performed by research staffs blinded to the treatment allocation. Second, all participants will come from one and the same medical university, which may decrease the sample representativeness.

In summary, this is the first randomized controlled trial to systematically evaluate the effectiveness of Baduanjin exercise for the physical and mental health of a college student population. If our study demonstrates a significant intervention effect, this would provide a rigorous evidence for the application of Baduanjin exercise among a college student population.

## Trial status

Recruitment started while the manuscript was being finished.

## Competing interests

The authors declare that they have no competing interests.

## Authors’ contributions

CLD, TJ, and ZGH conceived of the study, designed the study protocol, and drafted the manuscript. LMY wrote the manuscript. ZGH revised study protocols and wrote several sections of the manuscript. TJ is in charge of coordination and direct implementation. YXH, LQ, LXL, ZX, LJZ, CB, and FQY helped to develop the study measures and analyses. All authors contributed to drafting the manuscript and have read and approved the final manuscript.
